# Genome-resolved gut metagenomics identifies an *Escherichia coli*-*Collinsella* signature associated with Wagner 4 gangrenous diabetic foot ulcers

**DOI:** 10.3389/fimmu.2026.1893357

**Published:** 2026-09-03

**Authors:** Liwei Li, Yanxia Luo, Shikai Liu, Chenghai Liang, Huaqiang Ruan, Peng Peng, Zhenxing Huang

**Affiliations:** 1 Department of Gastroenterology, The Second Affiliated Hospital of Guangxi Medical University, Nanning, China; 2 Department of Endocrinology, The First Affiliated Hospital of Guangxi Medical University, Nanning, China

**Keywords:** *Collinsella*, diabetic foot ulcer, *Escherichia coli*, gut microbiome, metagenome-assembled genomes

## Abstract

Diabetic foot ulcers (DFU) are a major complication of type 2 diabetes mellitus, but whether the gut microbiome captures systemic microbial features associated with advanced ulcer severity remains unclear. We performed shotgun metagenomic sequencing of stool samples from 43 patients with type 2 diabetes mellitus and active DFU, comparing Wagner grades 1–3 (*n* = 30) with Wagner 4 gangrenous disease (*n* = 13). *De novo* assembly and binning recovered 440 dereplicated metagenome-assembled genomes (MAGs) meeting medium-quality or high-completeness/low-contamination thresholds. Community-level diversity and dominant-taxon composition did not separate Wagner 4 from Wagner 1–3, indicating that advanced disease was not reflected by broad ecological restructuring. Feature-level analysis instead identified a genome-resolved MAG profile. To prioritize robust candidates, we combined two complementary approaches: random forest (RF) stability selection, which identified 24 MAGs with reproducibly high classification importance across resampled folds, and covariate-adjusted MaAsLin2 differential-abundance testing. Intersecting the results of both approaches prioritized three MAGs supported by each method: one *Escherichia coli* MAG enriched in Wagner 4 and two *Collinsella* MAGs depleted in Wagner 4. This three-MAG signature (out-of-bag AUC = 0.703) retained much of the discriminatory information captured by the broader 24-MAG RF classifier, with concordant, opposing abundance directions across classifier interpretation, differential-abundance testing, and per-MAG abundance distributions. Functional annotation further separated the Wagner 4-enriched *Escherichia coli* from the *Collinsella* MAGs. The *Escherichia coli* MAG carried antibiotic-resistance and virulence-factor signals and encoded respiratory metabolic capacity, whereas the two *Collinsella* MAGs lacked detectable resistance and virulence hits and showed metabolically compact profiles. Exploratory clinical association analysis linked the *E. coli*–*Collinsella* abundance score to longer DFU duration, consistent with a gut microbial correlate of chronic or advanced disease burden. These findings support longitudinal gut metagenomic validation to determine whether this signal tracks DFU progression, treatment response, or recovery.

## Graphical Abstract

**Figure:**
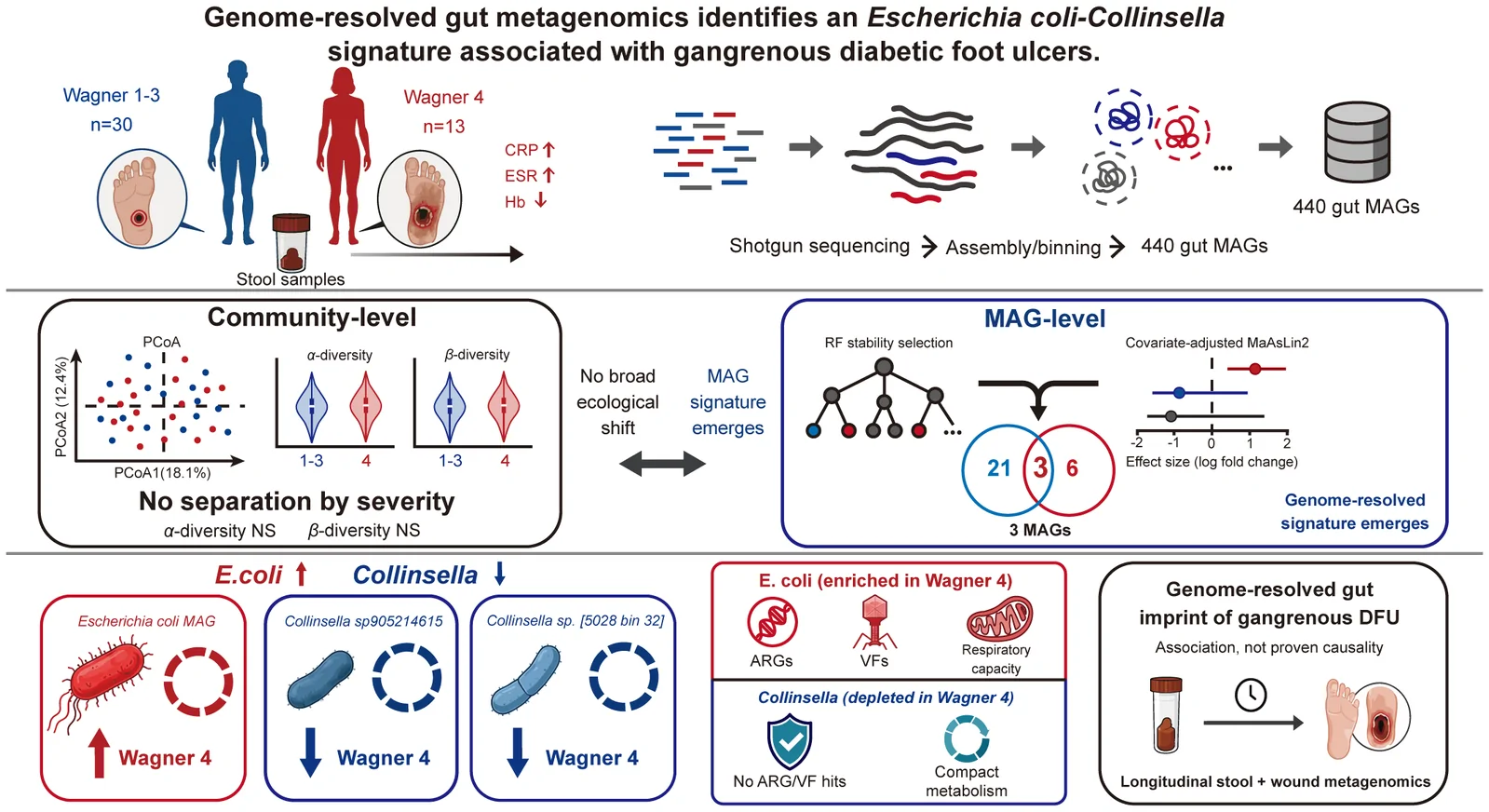

## Highlights

- Genome-resolved stool metagenomics identified a gut MAG signature associated with Wagner 4 gangrenous diabetic foot ulcers.
- Wagner 4 was marked by *Escherichia coli* enrichment and coordinated depletion of multiple *Collinsella* MAGs.
- The *E. coli* MAG carried resistance and virulence signals, whereas the *Collinsella* MAGs showed metabolically compact profiles.

## Introduction

Diabetic foot ulceration is one of the most consequential complications of type 2 diabetes mellitus (T2DM), affecting approximately 6% of people with diabetes worldwide and carrying a high risk of recurrence, infection, hospitalization, and lower-limb amputation (1, 2). More broadly, gut microbial alterations have been linked to metabolic and inflammatory disease in population and multi-omic studies (3, 4). Clinical severity is usually defined by wound depth, tissue loss, infection, and ischemia, and advanced disease is accompanied by systemic inflammation and poor outcomes (2, 5). However, the biological features that distinguish patients with more severe diabetic foot ulcers (DFU) remain incompletely defined beyond local wound assessment and conventional clinical markers.

Microbiological studies of DFU have largely focused on the ulcer site. Culture-based surveys and meta-analyses have established diabetic foot infection as a heterogeneous, often polymicrobial condition (5, 6), and sequencing-based studies have shown that the wound microbiome varies with ulcer depth, glycemic control, microbial diversity, strain-level variation, and clinical outcome (7, 8). These data support a role for local microbial communities in DFU biology but do not by themselves address whether the gut microbiome captures systemic microbial features associated with ulcer severity. A complementary conceptual framework, the gut-skin axis hypothesis, proposes that gut microbiota and their metabolites can influence distal skin and wound biology through circulating immune and metabolic signals and has been specifically invoked for diabetic wound healing (27); this hypothesis motivates asking whether gut-level microbial features, rather than only wound-site communities, track DFU severity.

Early evidence also suggests that the intestinal microbiota may differ in patients with DFU. A 16S rRNA-based analysis of public gut-microbiome datasets reported enrichment of intestinal Streptococcus in patients with DFU relative to diabetic patients without ulceration and proposed a contribution of this genus to DFU pathogenesis (9). However, this evidence was limited to amplicon-based, genus-level profiling and could not resolve individual genomes or link taxonomic signals to functional potential.

Genome-resolved shotgun metagenomics provides an opportunity to move beyond community-level diversity metrics and genus-level summaries. Metagenome-assembled genomes (MAGs) can distinguish closely related lineages and link abundance patterns to functional potential, including antibiotic-resistance genes, virulence factors, and metabolic pathways. Large-scale human microbiome studies have shown that metagenomic assembly recovers underrepresented microbial diversity and improves the interpretability of gut metagenomes (10, 11). This resolution is particularly relevant for DFU, where strain- and species-level variation in wound microbiota has been associated with clinical outcomes (8).

Here, we investigated whether gut MAGs are associated with DFU severity in 43 patients with T2DM and active DFU, comparing Wagner 1–3 with Wagner 4. We reconstructed a genome-resolved gut MAG catalog, assessed community diversity and composition, identified stable severity-associated MAGs using random forest (RF) stability selection, tested abundance associations with covariate-adjusted MaAsLin2 models, and profiled candidate MAGs for resistance, virulence, and metabolic potential. This design allowed us to ask whether advanced DFU severity is reflected by a gut microbial signal that is not apparent in overall diversity but emerges at genome-resolved feature and functional levels.

## Results

To evaluate whether gut microbial features track DFU severity, we first compared clinical and inflammatory characteristics between Wagner 1–3 and Wagner 4 patients, then tested whether standard community-level diversity metrics separated the two groups. We next combined RF stability selection with covariate-adjusted MaAsLin2 differential-abundance testing to identify genome-resolved MAG-level signals, intersected the two approaches to prioritize a minimal robust MAG set, and finally characterized the functional potential and clinical correlates of the prioritized MAGs.

### Cohort characteristics showed greater inflammatory burden in Wagner 4

To characterize the cohort and identify potential confounders for downstream microbiome analyses, we first compared demographic, anthropometric, diabetes-related, and inflammatory variables between Wagner 1–3 and Wagner 4 patients. Stool shotgun metagenomes were generated from 43 patients with T2DM and active DFU, stratified by Wagner grade into Wagner 1–3 (*n* = 30; grades 1, 2, and 3) and Wagner 4 (*n* = 13). Age, BMI, HbA1c, and diabetes duration were similar between groups (all *P* > 0.4). By contrast, Wagner 4 patients had higher systemic inflammatory burden, including higher C-reactive protein [44.4 (25.2–100.0) vs. 5.0 (5.0–29.5) mg/L; P = 0.005], higher erythrocyte sedimentation rate (92 [68–105] vs. 58 [33–80] mm/h; *P* = 0.003), and lower hemoglobin (86.0 [82.0–100.9] vs. 116.5 [95.1–125.0] g/L; *P* = 0.022). Wagner 4 was also found more frequently in male patients (12/13, 92.3%) than in Wagner 1–3 (14/30, 46.7%; *P* = 0.013); this sex imbalance was therefore included as a covariate in the subsequent differential-abundance models (**Table 1**).

**Table 1:** Baseline characteristics of the study cohort by Wagner severity group.

Characteristic	Wagner 1–3 (*n* = 30)	Wagner 4 (*n* = 13)	*P*-value	Test
Demographics				
Age, years	62.0 [58.0, 67.0]	62.0 [61.0, 70.0]	0.496	Wilcoxon
Sex, *n* (%)			0.013	chi-square
Male	14 (46.7)	12 (92.3)		
Female	16 (53.3)	1 (7.7)		
Ethnicity, *n* (%)			0.460	Fisher
Han	23 (76.7)	8 (61.5)		
Minority	7 (23.3)	5 (38.5)		
Anthropometric				
Height, cm	161.6 ± 7.9	164.9 ± 4.8	0.096	t-test
Weight, kg	57.5 [52.3, 69.6]	64.0 [59.4, 73.0]	0.109	Wilcoxon
BMI, kg/m²	22.4 [21.0, 25.6]	23.0 [21.7, 25.7]	0.513	Wilcoxon
Diabetes/DFU history				
DM duration, years	9.0 [3.0, 14.5]	9.0 [5.0, 20.0]	0.427	Wilcoxon
DFU duration, days	30.0 [8.5, 52.5]	60.0 [15.0, 60.0]	0.161	Wilcoxon
Prior DFU history, *n* (%)			1.000	Fisher
No	23 (76.7)	10 (76.9)		
Yes	7 (23.3)	3 (23.1)		
HbA1c, %	8.5 [6.9, 9.8]	8.7 [6.4, 10.4]	1.000	Wilcoxon
Inflammatory markers				
Peak temperature, °C	36.8 [36.6, 37.0]	36.8 [36.7, 36.9]	0.800	Wilcoxon
WBC, ×10^9^/L	8.6 [6.6, 10.2]	10.6 [7.7, 12.5]	0.135	Wilcoxon
Neutrophil ratio, %	0.7 ± 0.1	0.7 ± 0.1	0.110	t-test
CRP, mg/L	5.0 [5.0, 29.5]	44.4 [25.2, 100.0]	0.005	Wilcoxon
PCT, ng/ml	0.06 [0.03, 0.55]	0.16 [0.06, 0.47]	0.126	Wilcoxon
ESR, mm/h	58.0 [33.0, 80.0]	92.0 [68.0, 105.0]	0.003	Wilcoxon
Biochemistry				
ALT, U/L	15.0 [11.0, 22.0]	17.0 [15.0, 28.0]	0.340	Wilcoxon
AST, U/L	17.0 [13.0, 22.0]	19.0 [16.0, 32.0]	0.470	Wilcoxon
Albumin, g/L	35.9 ± 8.3	31.9 ± 6.1	0.087	t-test
Urea, mmol/L	6.1 [5.0, 8.7]	7.4 [5.1, 9.8]	0.341	Wilcoxon
Creatinine, µmol/L	83.5 [68.2, 109.2]	108.0 [81.0, 132.0]	0.116	Wilcoxon
Hemoglobin, g/L	116.5 [95.1, 125.0]	86.0 [82.0, 100.9]	0.022	Wilcoxon
Platelets, ×10^9^/L	320.7 ± 128.3	383.6 ± 179.6	0.268	t-test
Total cholesterol, mmol/L	4.12 [3.39, 5.17]	3.31 [2.79, 3.41]	0.111	Wilcoxon
Triglycerides, mmol/L	1.15 [0.86, 1.78]	1.14 [0.88, 1.36]	0.817	Wilcoxon
HDL-C, mmol/L	0.95 ± 0.30	0.90 ± 0.29	0.589	t-test
LDL-C, mmol/L	2.41 ± 0.84	1.82 ± 0.98	0.076	t-test

Continuous variables: mean ± SD (Welch’s t-test) if normally distributed (Shapiro-Wilk *p* ≥ 0.05 in both groups), otherwise median [Q1, Q3] (Wilcoxon rank-sum test).

Categorical variables: *n* (%); Fisher exact test (any expected cell < 5) or chi-square test.

CRP values reported as <10 mg/L by the laboratory were imputed at 5 mg/L (limit-of-detection midpoint); one value reported as >140 mg/L was set to 140 mg/L.

DM, diabetes mellitus; DFU, diabetic foot ulcer; HbA1c, glycated hemoglobin; WBC, white blood cell count; CRP, C-reactive protein; PCT, procalcitonin; ESR, erythrocyte sedimentation rate.

### Community-level gut microbiome profiles did not separate Wagner 4 from Wagner 1–3

We next asked whether these clinical differences were mirrored by broad shifts in gut microbial community structure. *De-novo* assembly and binning of metagenomic reads yielded 440 dereplicated MAGs meeting medium-quality or high-completeness/low-contamination thresholds, of which 410 (93.2%) were classified to the species level by GTDB-Tk (**Supplementary Table 1**). The catalog spanned 11 phyla and was dominated by *Bacillota* (*n* = 307; 69.8%), *Bacteroidota* (*n* = 55; 12.5%), *Actinomycetota* (*n* = 42; 9.5%), and *Pseudomonadota* (*n* = 17; 3.9%), consistent with the broad phylum structure of human gut metagenomes.

**Figure 1:**
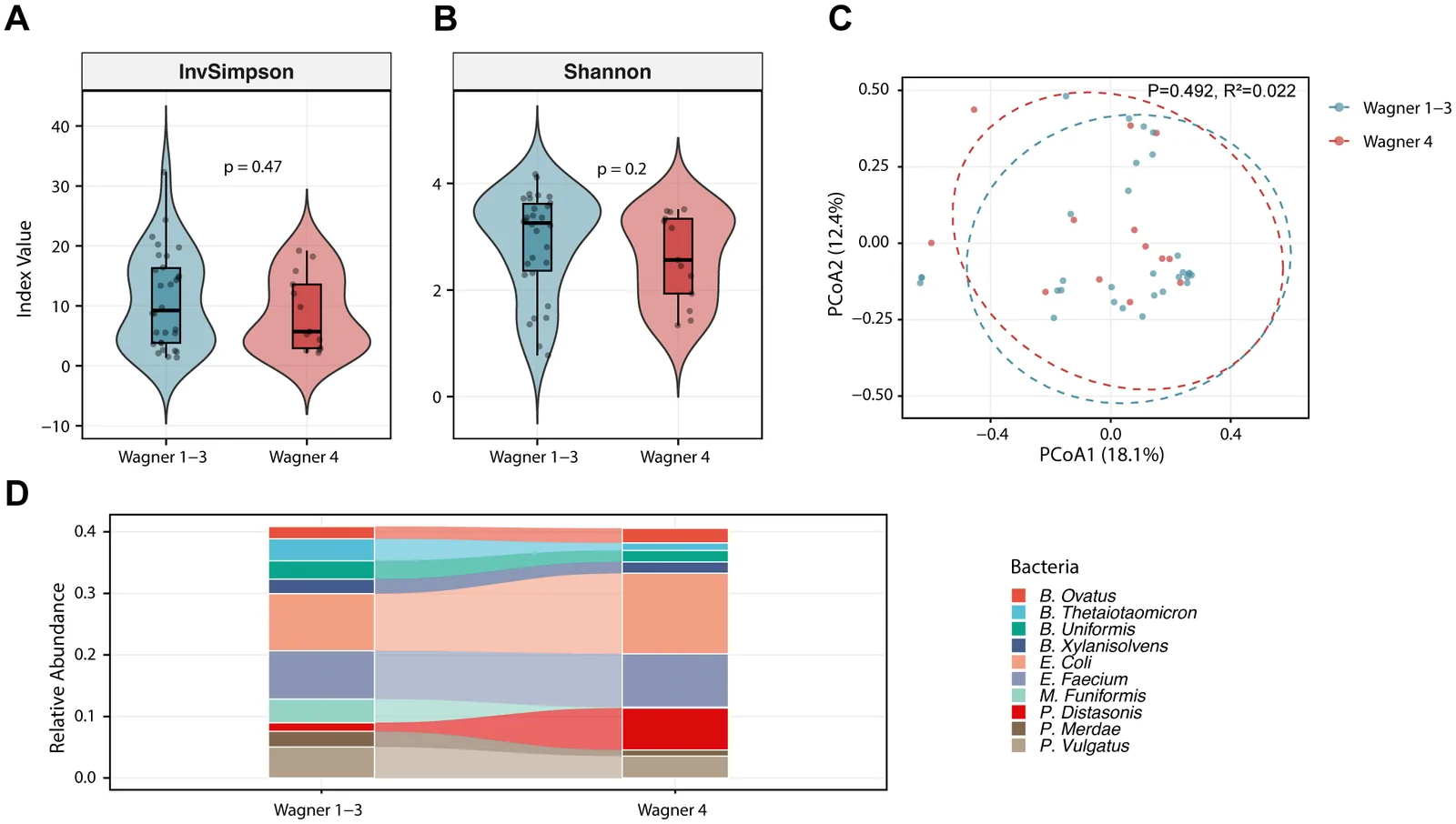
Cohort overview and community-level microbiome profiles. **(A, B)** Violin and box plots of **(A)** Inverse Simpson and **(B)** Shannon α-diversity in Wagner 1–3 (*n* = 30) and Wagner 4 (*n* = 13); each point denotes one sample. *P*-values are from two-sided Wilcoxon rank-sum tests. **(C)** Bray-Curtis principal coordinate analysis (PCoA), with points colored by Wagner group and dashed ellipses indicating 95% confidence regions. The *P*-value and R² are from PERMANOVA (adonis2, 999 permutations). **(D)** Sankey-style composition map of the ten most abundant gut species across groups. Ribbon widths are proportional to mean within-group relative abundance. Wagner 1–3, blue; Wagner 4, red.

Conventional community-level metrics showed little separation by DFU severity. Neither Inverse Simpson nor Shannon α-diversity differed between groups (Wilcoxon *P* = 0.47 and *P* = 0.20, respectively; **Figure 1A, B**), and Bray-Curtis β-diversity ordination showed substantial overlap, with no significant global structure by PERMANOVA (*R*² = 0.022, *P* = 0.492; **Figure 1C**). A Sankey-style map of the ten most abundant MAGs showed only modest compositional reorganization, most notably an apparent expansion of *Escherichia coli* in Wagner 4 against largely stable *Bacteroides* and *Parabacteroides* species (**Figure 1D**). Thus, severity-associated microbial variation was not captured by total diversity or dominant-taxon structure.

### A stable gut MAG signature distinguished Wagner 4 from Wagner 1–3

**Figure 2:**
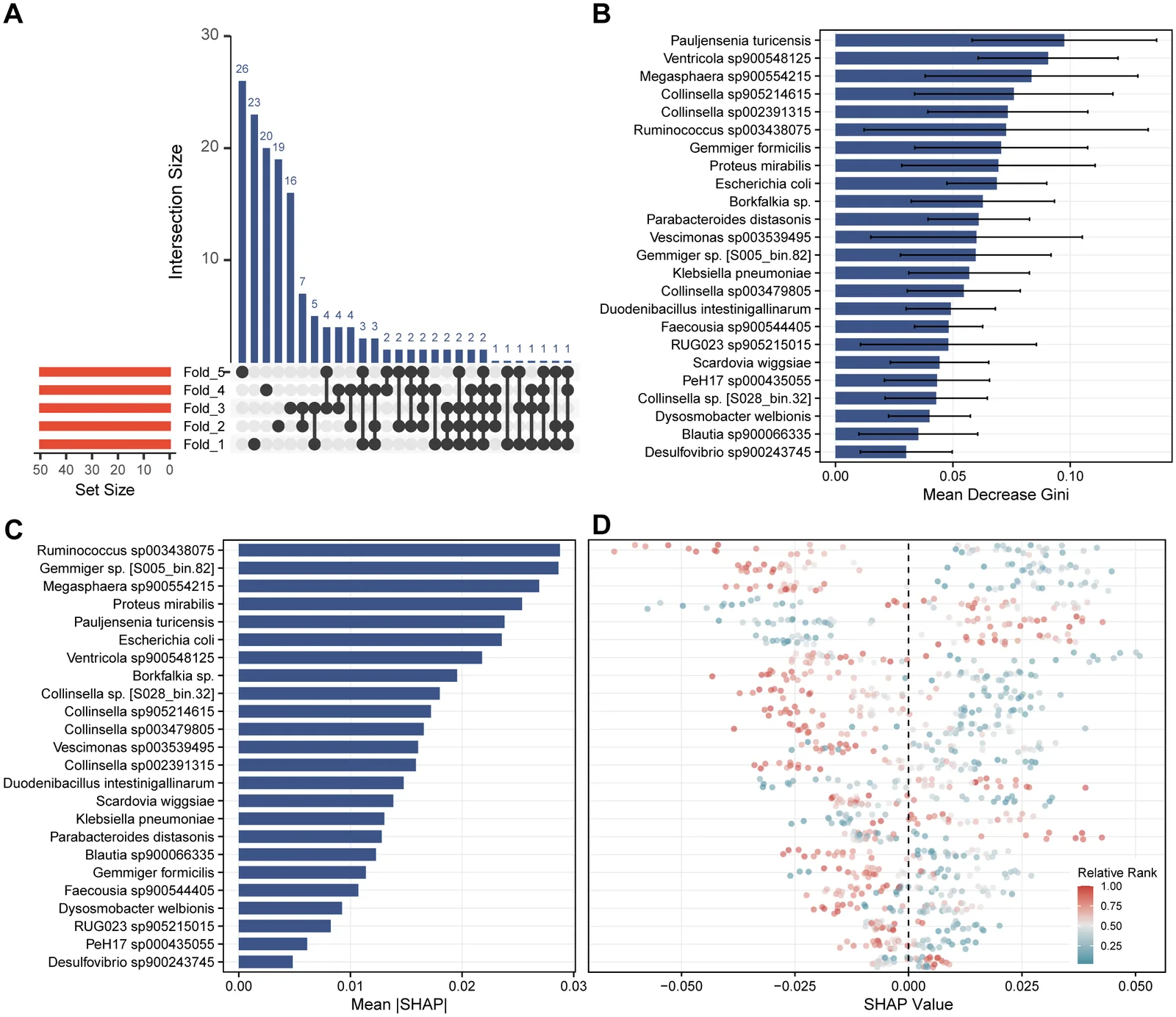
Random forest identified 24 RF-selected MAGs. **(A)** UpSet plot showing the intersection of MAGs selected across the five RF folds; vertical bars indicate the number of MAGs shared by each fold combination. **(B)** Mean decrease in Gini importance for the 24 MAGs selected by the class-balanced random forest in at least three of five cross-validation folds. Bars show means across folds, with error bars indicating across-fold s.d. **(C)** Mean absolute SHAP values for the RF-selected MAGs, summarizing each MAG’s contribution to classifier output. **(D)** Per-sample SHAP values for each RF-selected MAG; points denote samples and are colored by within-MAG abundance rank (red, high; blue, low). Positive SHAP values indicate a shift toward Wagner 4, and negative values indicate a shift toward Wagner 1–3.

To identify MAGs whose predictive importance was robust to how patients were partitioned into training folds—that is, repeated random splits of the 43 patients into a subset used to train the classifier and a held-out subset used to test it, a standard cross-validation procedure for guarding against overfitting—rather than reflecting the composition of any single split, we performed RF stability selection: the classifier was retrained across five such resampled folds, and only MAGs selected as important in at least three of the five folds were retained for downstream analysis. This procedure yielded 24 RF-selected MAGs with heterogeneous importance values, indicating that the retained feature set captured a broader stability signal rather than only the top-ranked RF features (**Figure 2A, B**; **Supplementary Table 2**). The selected MAGs included *E. coli*, *Klebsiella pneumoniae*, and *Proteus mirabilis*, which have been reported in diabetic foot infections (6), together with gut-associated lineages including *Collinsella* spp., *Gemmiger* spp., and *Ruminococcus sp003438075*.

To interpret how each RF-selected MAG contributed to classifier predictions, we calculated SHAP (SHapley Additive exPlanations) values, which quantify the direction and magnitude by which a given MAG’s abundance shifts an individual sample’s predicted probability of belonging to Wagner 4 relative to Wagner 1–3. This analysis revealed opposing classifier directions among the RF-selected MAGs (**Figure 2C, D**). High *E. coli* abundance shifted predictions toward Wagner 4, whereas high abundances of *Ruminococcus sp003438075*, *Collinsella sp905214615*, *Collinsella sp002391315*, *Collinsella sp003479805*, and *Collinsella* sp. *[S028_bin.32]* shifted predictions toward Wagner 1–3. Their abundance distributions were heterogeneous across MAGs and Wagner groups (**Supplementary Figure 1**).

### Wagner 4 was marked by *E. coli* enrichment and *Collinsella* MAG depletion across taxonomic levels

**Figure 3:**
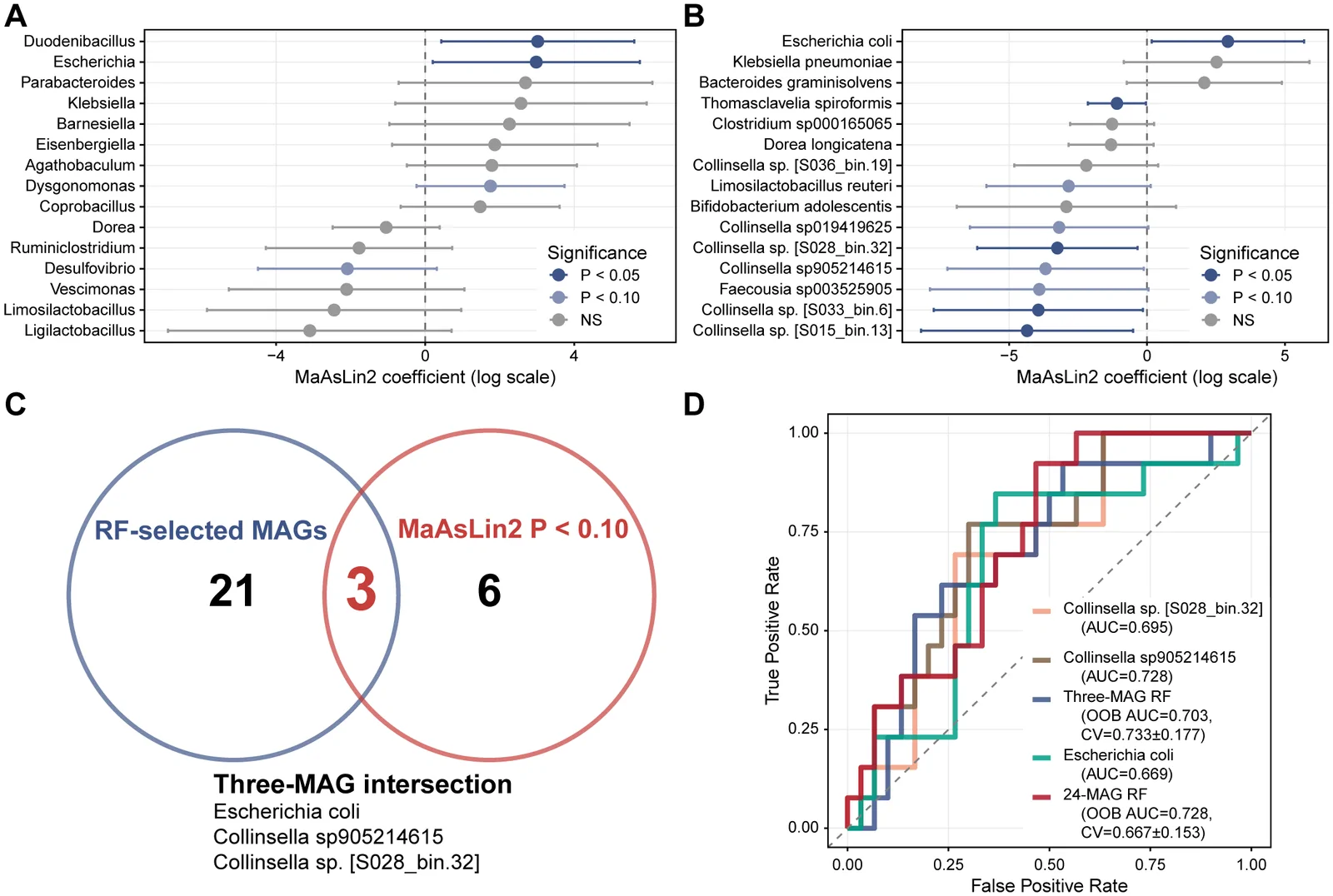
Cross-method prioritization identified an *E. coli*–*Collinsella* three-MAG signal in Wagner 4. **(A)** MaAsLin2 forest plot at genus level, showing coefficients (log scale) with 95% CIs for Wagner 4 versus Wagner 1–3 after adjustment for age, sex, BMI, and HbA1c. **(B)** MaAsLin2 forest plot at species/MAG-resolved level, otherwise as in (**A**). Dot colors indicate significance: dark blue, *P* < 0.05; light blue, *P* < 0.10; gray, not significant. **(C)** Venn diagram showing the intersection between the RF-selected MAGs and MAGs reaching *P* < 0.10 in MaAsLin2 with concordant direction between unadjusted and adjusted models. The overlap yielded three MAGs. **(D)** Receiver-operating-characteristic comparison between the 24-MAG and three-MAG RF models; AUCs are out-of-bag estimates, and the *P*-value is from DeLong’s test.

**Figure 4:**
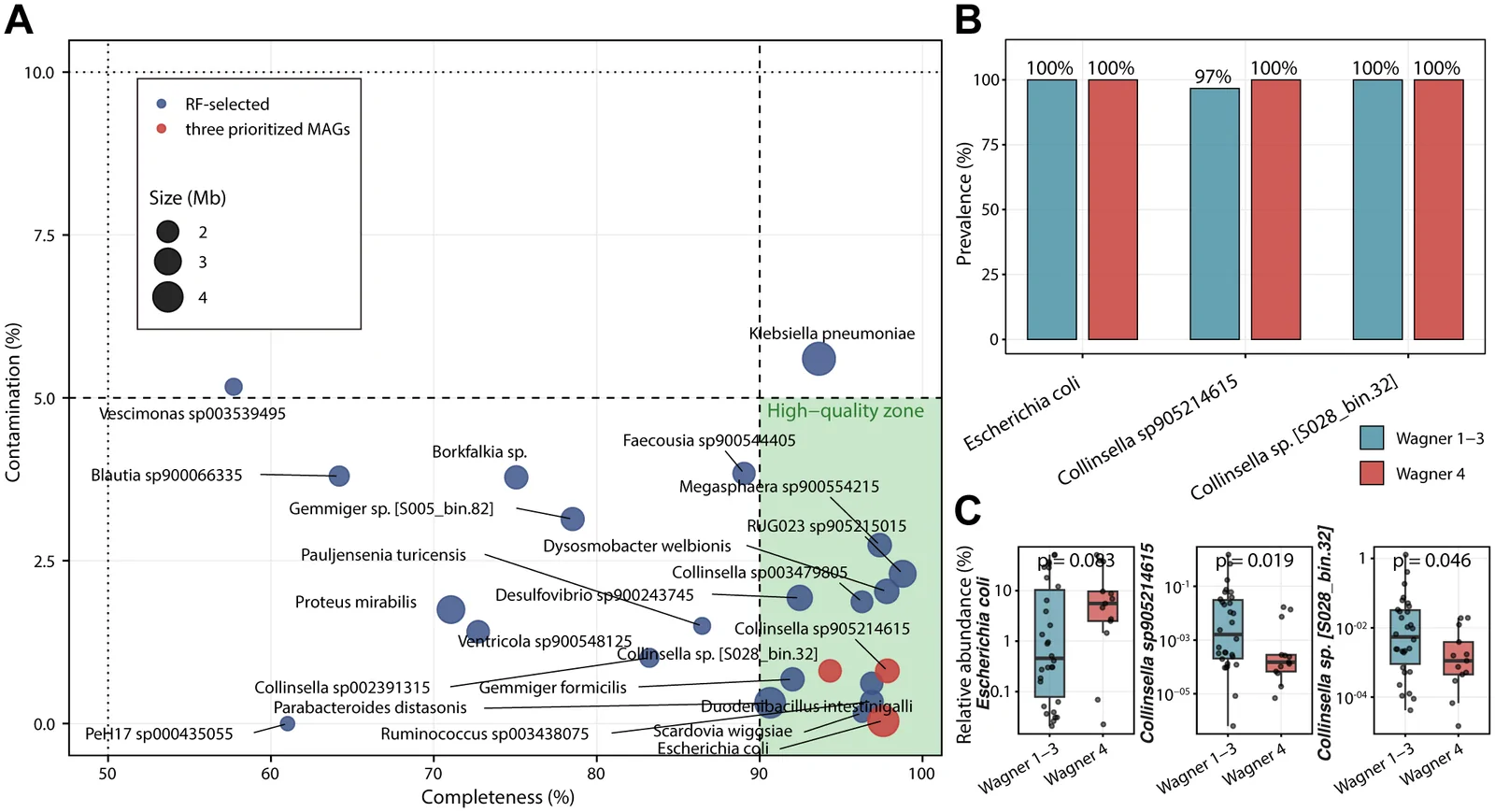
The three prioritized MAGs showed high completeness and low contamination with divergent prevalence and abundance profiles. **(A)** CheckM completeness versus contamination for the RF-selected MAGs; circle size is proportional to genome size (Mb), red points mark the three prioritized MAGs, and blue points mark the remaining RF-selected MAGs. The shaded zone indicates the MIMAG-derived completeness/contamination thresholds commonly used for high-completeness, low-contamination draft MAGs (completeness ≥ 90% and contamination < 5%). **(B)** Prevalence of the three prioritized MAGs in Wagner 1–3 and Wagner 4. **(C)** Relative-abundance distributions of these MAGs by Wagner group. *P*-values are from two-sided Wilcoxon rank-sum tests.

MaAsLin2 (Microbiome Multivariable Associations with Linear Models) tests each taxon’s or MAG’s abundance for association with a variable of interest, here Wagner grade, while adjusting for covariates; we used it as an orthogonal, hypothesis-testing complement to the RF stability selection above. Covariate-adjusted MaAsLin2 analysis at genus, species, and MAG levels recovered a consistent *E. coli*/*Collinsella* contrast (**Supplementary Table 3**). At genus level, *Duodenibacillus* (coefficient +3.02, *P* = 0.028) and *Escherichia* (+2.98, *P* = 0.043) were enriched in Wagner 4 (**Figure 3A**). The *E. coli* signal persisted at both species and MAG levels (+2.93, *P* = 0.045), whereas *Thomasclavelia spiroformis* was depleted (−1.09, *P* = 0.050). Three additional *Collinsella* MAGs were depleted in Wagner 4: *Collinsella* sp. *[S015_bin.13]* (−4.35, *P* = 0.033), *Collinsella* sp. *[S028_bin.32]* (−3.25, *P* = 0.035) and *Collinsella* sp. *[S033_bin.6]* (−3.94, *P* = 0.049) (**Figure 3B**). More broadly, 8 of 9 *Collinsella* MAGs detected in the catalog had negative Wagner 4 coefficients, and 5 reached *P* < 0.10 (**Supplementary Table 3**). Together, these results identified Wagner 4 as a state marked by *Escherichia*/*E. coli* enrichment and broad *Collinsella* depletion (**Figures 3A**, **4B**; **Supplementary Table 3**).

### An *E. coli*-enriched and *Collinsella*-depleted three-MAG signal captured Wagner 4

To distill the RF- and MaAsLin2-based signals into a minimal, reproducible feature set, we intersected the two lists. Intersecting the RF-selected MAGs with MAG-level MaAsLin2 hits at *P* < 0.10 and directionally consistent effects between unadjusted and covariate-adjusted models yielded three MAGs shared by both approaches: *Escherichia coli* (Wagner 4-enriched, *P* = 0.045), *Collinsella sp905214615* (Wagner 4-depleted, *P* = 0.050), and *Collinsella* sp. *[S028_bin.32]* (Wagner 4-depleted, *P* = 0.035) (**Figure 3C**; **Supplementary Table 4**). All three showed high completeness and low contamination by CheckM estimates (completeness 94.3%–97.8%, contamination ≤ 0.81%; **Figure 4A**; **Supplementary Table 5**).

The three-MAG RF model retained much of the discriminatory information in the 24-MAG RF-selected model. Its out-of-bag (OOB) AUC was 0.703 (5-fold cross-validation AUC 0.733 ± 0.177), comparable to the 24-MAG RF model (OOB AUC 0.728; fivefold CV AUC 0.667 ± 0.153) under the same training protocol (**Figure 3D**). The two RF models showed statistically comparable OOB AUCs (DeLong ΔAUC = 0.026, *P* = 0.78; **Supplementary Table 6**). At the single-MAG level, the *E. coli*–*Collinsella* trio had the highest median AUC across candidate categories (median AUC = 0.70), whereas RF-only candidates were more variable, and MaAsLin2-only candidates had lower median AUCs (**Supplementary Figure 2**; **Supplementary Table 7**).

### The three-MAG signal reflected *E. coli* expansion and *Collinsella* depletion

We next asked whether this three-MAG signal reflected presence/absence differences between groups or, instead, shifts in abundance among taxa prevalent in both groups. All three MAGs were highly prevalent in both groups (**Figure 4B**; **Supplementary Table 8**). *E. coli* and *Collinsella* sp. *[S028_bin.32]* were detected in 100% of patients, whereas *Collinsella sp905214615* was detected in 96.7% of Wagner 1–3 and 100% of Wagner 4 samples (*P* = 1.0, Fisher’s exact test). Despite this similar prevalence, abundance distributions diverged. *E. coli* tended to be more abundant in Wagner 4 than in Wagner 1–3 (median 5.56% vs. 0.46%; Wilcoxon *P* = 0.083), whereas both *Collinsella* MAGs were significantly lower in Wagner 4 (*Collinsella sp905214615*, 0.0001% vs. 0.0017%, *P* = 0.019; *Collinsella* sp. *[S028_bin.32]*, 0.0011% vs. 0.0056%, *P* = 0.046). Thus, the three-MAG signal reflected abundance redistribution among highly prevalent taxa rather than the presence or absence of a severity-specific organism.

### Functional profiling linked the Wagner 4-enriched *E. coli* MAG to virulence-factor enrichment and respiratory metabolic capacity

**Figure 5:**
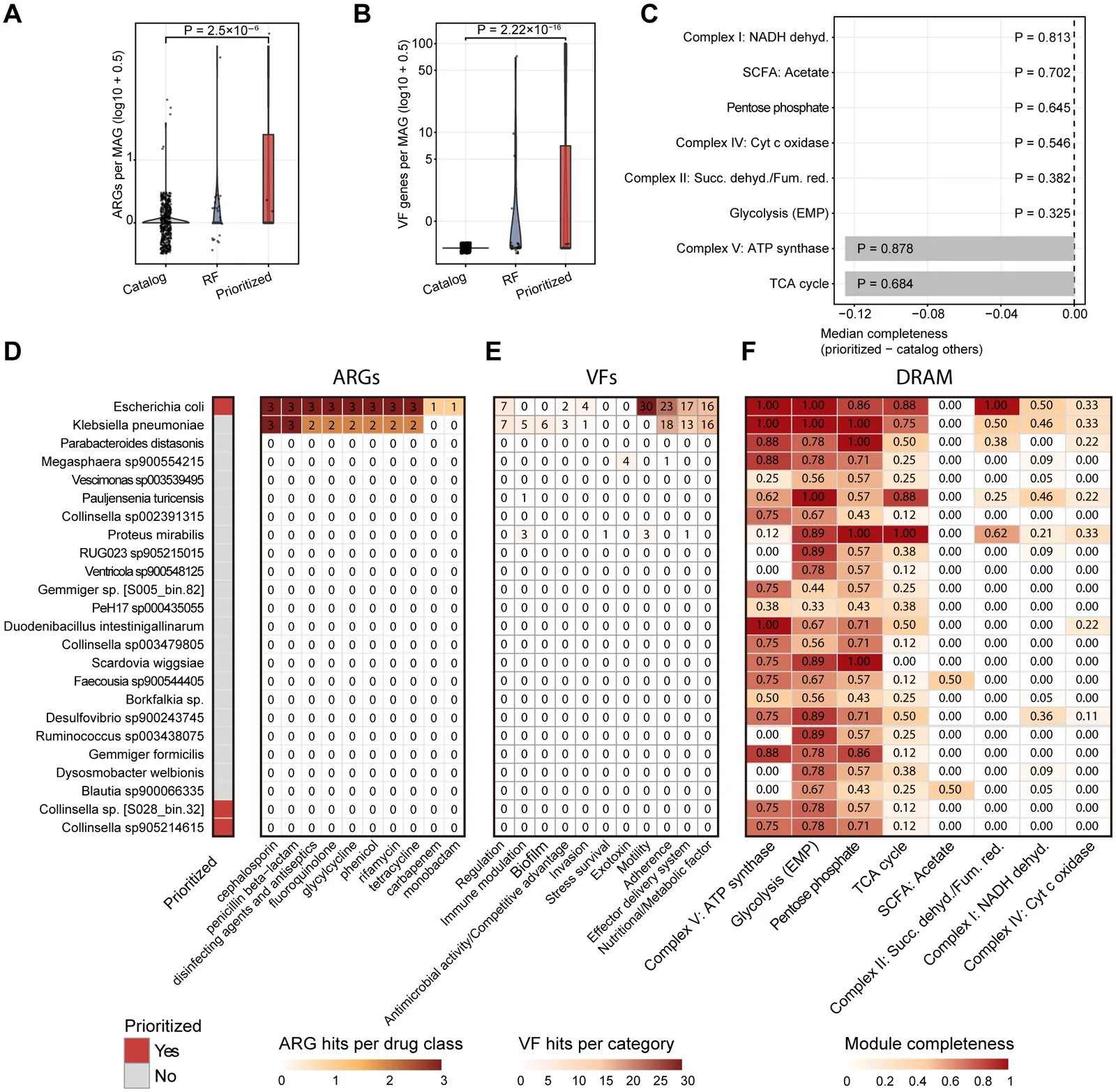
Functional profiles of RF-selected MAGs and the three prioritized MAGs. **(A)** Per-MAG antibiotic-resistance gene (ARG) load across all other catalog MAGs, RF-selected MAGs outside the three-MAG intersection, and the three prioritized MAGs. Values are RGI Perfect/Strict hits plotted as log10 (count + 0.5); the displayed *P*-value is a descriptive two-sided Wilcoxon rank-sum comparison of the three MAGs versus all other catalog MAGs. **(B)** Per-MAG virulence-factor (VF) load based on ABRicate-VFDB hits, plotted as log10(count + 0.5), with the displayed *P*-value calculated as in **(A)**. **(C)** Per-MAG DRAM theme completeness, summarized as the mean module-completeness score across selected metabolic themes; the displayed *P*-value is a descriptive comparison as in **(A)**. **(D)** Heat map of RGI Perfect/Strict ARG hits across the RF-selected MAGs. Cell values show the number of ARG hits assigned to each CARD drug class. **(E)** Heat map of VFDB virulence-factor counts across the RF-selected MAGs. Cell values show the number of VF hits assigned to each VFDB category. **(F)** Heat map of selected DRAM metabolic-theme completeness across the RF-selected MAGs. Cell values range from 0 to 1 and indicate module completeness.

Functional annotation of the MAG catalog linked the E. coli–Collinsella signal to contrasting patterns of antibiotic resistance, virulence, and metabolic profiles (**Figure 5**; **Supplementary Tables 9**–**13**). We first summarized per-MAG antibiotic-resistance gene (ARG), virulence factor (VF), and DRAM (Distilled and Refined Annotation of Metabolism) metabolic-theme features across the RF-selected MAGs and then used catalog-level comparisons only as descriptive context.

ARG content was concentrated in two Pseudomonadota members among the RF-selected MAGs. The Wagner 4-enriched *E. coli* MAG carried three RGI hits spanning eight core CARD drug classes (cephalosporin, penicillin-β-lactam, fluoroquinolone, tetracycline, glycylcycline, phenicol, rifamycin, and disinfecting agents and antiseptics), with one hit additionally annotated to carbapenem and monobactam resistance (**Figure 5D**; **Supplementary Table 9**). The RF-selected *K. pneumoniae* MAG carried three RGI hits spanning the same eight core drug classes but lacked the carbapenem and monobactam annotations (**Figure 5D**; **Supplementary Table 9**). No other RF-selected MAG carried an RGI-detected resistance gene, indicating that the ARG signal among the three MAGs was restricted to *E. coli* rather than shared by all three (**Figure 5A, D**; **Supplementary Table 9**).

Virulence-factor content was even more concentrated in the same *E. coli* MAG. *E. coli* encoded 99 VFs across seven VFDB functional categories, with the largest contributions from motility (*n* = 30), adherence (*n* = 23) and effector-delivery system (*n* = 17), predominantly functions related to host colonization (**Figure 5E**; **Supplementary Tables 10**, **11**). *K. pneumoniae* encoded 69 VFs with similar category coverage, whereas *Proteus mirabilis*, *Megasphaera sp900554215*, and *Pauljensenia turicensis* encoded 8, 5, and 1 VFs, respectively. The two Wagner 4-depleted *Collinsella* MAGs carried no VFDB hits at the defined threshold, whereas VF content among RF-selected MAGs was concentrated mainly in *E. coli* and *K. pneumoniae*. VF detection should therefore be interpreted as pathogen-like gene content in the Wagner 4-enriched *E. coli* MAG, rather than as a complete census of host-interaction functions across the catalog.

DRAM theme analysis further separated the Wagner 4-enriched *E. coli* MAG from the two Wagner 4-depleted *Collinsella* MAGs (**Figure 5F**; **Supplementary Table 12**). The two *Collinsella* MAGs both encoded modules for F-type ATP synthase (Complex V module completeness 0.75), glycolysis (EMP 0.78), and the pentose phosphate pathway (0.57–0.71), but lacked respiratory complexes II and IV, had limited TCA-cycle completeness (0.125), and contained no detectable SCFA-acetate biosynthetic genes. By contrast, the Wagner 4-enriched *E. coli* MAG showed higher completeness for glycolysis (1.00), the pentose phosphate pathway (0.86), Complex II succinate dehydrogenase/fumarate reductase (1.00), the TCA cycle (0.88), and partial Complex IV cytochrome c oxidase (0.33), consistent with encoded facultative-anaerobic respiratory capacity. Given the small three-MAG set, DRAM patterns were interpreted descriptively at the per-MAG level; a catalog-level comparison of the three MAGs with the remaining 416 catalog MAGs did not identify any enriched theme (minimum *P* = 0.325; **Figure 5C**; **Supplementary Table 13**).

Taken together, Wagner 4 was characterized in this cohort by an inverse abundance-functional pattern: expansion of a virulence-factor-rich, respiration-competent *E. coli* MAG and depletion of two metabolically compact *Collinsella* MAGs. Across the full set of RF-selected MAGs, the *E. coli*−*Collinsella* signal aligned opposite abundance directions with contrasting ARG/VF burdens and DRAM metabolic features (**Supplementary Figure 3**).

### The three-MAG abundance profile was associated with DFU duration

Having established a genome-resolved and functional signature distinguishing Wagner 4, we finally asked whether this signal was associated with clinical measures of disease burden. Across the 43-patient cohort, a composite *E. coli*-*Collinsella* abundance score, reflecting higher *E. coli* abundance together with lower abundance of *Collinsella sp905214615* and *Collinsella* sp. *[S028_bin.32]* was positively correlated with DFU duration (Spearman *ρ* = 0.50, *P* = 5.76 × 10^-4^, BH *q* = 0.013; **Supplementary Figure 4**; **Supplementary Table 14**). At the single-MAG level, *E. coli* showed the same direction of association with DFU duration (*ρ* = 0.37, *P* = 0.016), whereas *Collinsella* sp. *[S028_bin.32]* and *Collinsella sp905214615* showed inverse associations (*ρ* = −0.39, *P* = 0.010 and *ρ* = −0.31, *P* = 0.044, respectively; **Supplementary Table 14**).

These associations were broadly retained after adjustment for age, sex, BMI, and HbA1c. In the primary adjusted model, the *E. coli*–*Collinsella* abundance score showed a nominal association with longer DFU duration (β = 0.46, *P* = 0.003, BH *q* = 0.062), consistent with trend-level evidence after multiple-testing adjustment; *E. coli* and *Collinsella* sp. *[S028_bin.32]* retained concordant positive and negative effect directions, respectively (**Supplementary Table 15**). The *E. coli*–*Collinsella* abundance score also remained nominally associated with DFU duration after additional adjustment for Wagner group (β = 0.48, *P* = 0.005; **Supplementary Table 15**). Among the top-ranked pooled pairwise associations, DFU duration and renal or hematological markers accounted for the largest MAG-clinical correlations (**Supplementary Figure 5**; **Supplementary Table 14**).

## Discussion

In this genome-resolved gut metagenomic study of patients with DFU, the clearest microbial signal distinguished Wagner 4 from Wagner 1–3 rather than tracking global community diversity. This distinction matters biologically: the Wagner system separates superficial ulcers, deeper soft-tissue or bone involvement, local gangrene, and more extensive gangrene (13). The signature identified here should therefore be interpreted primarily as a candidate feature of advanced, gangrenous, or ischemic DFU, not as evidence for a smooth microbiome gradient across all Wagner grades. The pattern was observed across RF stability selection, SHAP-based directionality, MaAsLin2 differential-abundance modeling, cross-method feature prioritization, functional annotation, and exploratory clinical association analysis (**Figures 1**–**5**; **Supplementary Figures 4** and **5**).

Most microbiological work in DFU has focused on infected ulcers, as reflected in current diabetic-foot-infection guidance (5) and culture-based meta-analyses (6). Sequencing studies have linked wound microbial composition with ulcer depth and clinical factors (7), whereas strain- and species-level metagenomics has connected wound communities with healing trajectories and therapeutic response (8). Functional metagenomic analyses have also associated antibiotic-resistance, biofilm, and virulence-related genes with poor outcomes, including non-healing and amputation (8, 14). Our findings are therefore complementary to wound microbiome studies because they characterize a distinct ecological compartment that may record systemic metabolic, inflammatory, and disease-duration states relevant to advanced DFU. Future longitudinal designs that extend stool metagenomics with wound sampling where feasible will be well positioned to test strain-level links between gut and wound communities.

The lack of separation in α-diversity, β-diversity, and dominant-taxon composition does not conflict with the MAG-level signal (**Figure 1**). Community-level metrics summarize broad ecological structure and can remain stable when a small number of genome-resolved lineages shift in opposite directions. This is particularly relevant to DFU, because strain- and species-level wound microbiome variation has been linked to clinical outcomes and treatment response (8). More broadly, large human gut genome catalogs show that species-level labels often conceal substantial genome-level diversity (10, 11). The present data extend that principle to stool metagenomes in DFU: the discriminating signal emerged at the MAG and feature levels, where *E. coli* and *Collinsella* lineages showed opposing abundance directions (**Figures 2**–**4**; **Supplementary Table 3**).

The Wagner 4-enriched *E. coli* MAG had several features consistent with a pathobiont-like gut signal in Wagner 4. *E. coli* is a normal gut commensal in many hosts, but the species also contains pathotypes whose virulence factors can mediate intestinal and extraintestinal disease (15). In this cohort, however, the directionality is most parsimoniously read from the clinical context. Wagner 4 patients showed higher CRP and ESR and lower hemoglobin than Wagner 1–3 patients (**Table 1**), placing the gut signal against a background of systemic inflammation and chronic tissue injury. Experimental work shows that host-mediated intestinal inflammation can reduce obligate anaerobes and favor *Enterobacteriaceae* overgrowth (16), that host-derived nitrate can give *E. coli* a respiratory growth advantage in the inflamed gut (17), and that microbiota-activated PPAR-gamma signaling can restrain dysbiotic *Enterobacteriaceae* expansion by limiting luminal respiratory electron acceptors (18). Together, these mechanisms make the observed *E. coli* expansion and respiratory metabolic capacity biologically plausible. The accompanying ARG/VF burden strengthens the functional concern around this MAG but should be interpreted separately because acquired resistance genes are not directly explained by inflammation-driven respiratory advantage. The overall pattern supports interpretation of the *E. coli* signal as a gut imprint of advanced inflammatory disease, while future longitudinal sampling will be important for testing whether it also precedes or contributes to DFU progression.

The Collinsella signal was broader than the two prioritized MAGs alone. Eight of nine Collinsella MAGs had negative Wagner 4 coefficients in the MaAsLin2 analysis, and two were part of the three-MAG set (**Supplementary Table 3**). These two Collinsella MAGs were highly prevalent but substantially less abundant in Wagner 4, and they lacked the ARG/VF burden observed in the Wagner 4-enriched E. coli MAG (**Figures 4**, **5D, E**; **Supplementary Tables 8**–**10**). Their DRAM profiles encoded glycolysis, the pentose phosphate pathway, and F-type ATP synthase, but not complete acetate biosynthesis or a complete TCA cycle (**Figure 5F**; **Supplementary Table 12**). Thus, their depletion should not be framed simply as loss of canonical SCFA producers. A more cautious interpretation is contraction of metabolically compact, saccharolytic lineages within Collinsella in Wagner 4. The literature also argues against assigning a fixed beneficial or harmful role to Collinsella. Collinsella enrichment has been reported in symptomatic atherosclerosis (19) and rheumatoid arthritis, where it has been linked to inflammatory and gut-barrier phenotypes (22), and genomic and functional characterization of Collinsella aerofaciens isolates has directly linked strain-level carbohydrate metabolism and ethanol-production capacity to pro-inflammatory potential (28), underscoring that this genus’s functional consequences depend on strain-level metabolic content rather than genus identity alone. Dietary fiber, bile-acid metabolism, diabetes status, and metformin exposure can further shape Collinsella-associated patterns (20, 21, 23, 24). The meaningful point in this study is therefore not that Collinsella is universally protective, but that multiple Collinsella MAGs were coherently depleted in Wagner 4 at genome-level resolution.

The exploratory clinical analysis supports this downstream-imprint interpretation. The composite *E. coli*–*Collinsella* abundance score, defined by higher *E. coli* abundance and lower abundance of the two Wagner 4-depleted *Collinsella* MAGs was positively correlated with DFU duration and remained nominally associated after adjustment for age, sex, BMI, and HbA1c (**Supplementary Figure 4**; **Supplementary Tables 14**, **15**). This pattern is more consistent with a microbial correlate of chronic or advanced disease burden than with a stand-alone diagnostic marker. The *E. coli*–*Collinsella* abundance score therefore provides a candidate stratification feature for validation in larger longitudinal cohorts, where its relationship to progression, treatment response, and recovery can be assessed directly.

Several aspects strengthen the study. First, shotgun metagenomics and MAG reconstruction provided genome-resolved resolution beyond 16S rRNA profiling or genus-level summaries. Second, the candidate signal was supported by two orthogonal approaches, namely RF stability selection and covariate-adjusted differential-abundance modeling. Third, SHAP analysis, abundance distributions, genome-quality assessment, and functional annotation provided a coherent interpretation of the E. coli-Collinsella profile. Finally, excluding recent antibiotic exposure reduced one major source of microbiome perturbation in DFU studies, although unmeasured treatment history and hospital-related exposures may still have influenced the gut microbiome.

This study also has important limitations, and its findings should be read as hypothesis-generating evidence from a pilot-scale cohort rather than a validated diagnostic signature; the three-MAG signature is best treated as a prioritization candidate for confirmation in larger, adequately powered cohorts rather than as a definitive biomarker. It was single-center and cross-sectional, with a modest sample size and only 13 Wagner 4 patients, and the nominal MaAsLin2 signals should be viewed as prioritization evidence pending FDR-level validation in larger cohorts.

Sex imbalance is a particular concern: 12 of the 13 Wagner 4 patients were male, versus 14 of 30 in Wagner 1–3 (**Table 1**). We addressed this by including sex as a covariate in all MaAsLin2 and clinical-association models, and the *E. coli* and *Collinsella* effect directions were consistent between unadjusted and covariate-adjusted analyses (**Figure 3A, B**; **Supplementary Table 3**). To test directly whether this imbalance was driving the three-MAG signature, we additionally performed a male-restricted MaAsLin2 sensitivity analysis (14 Wagner 1–3 vs. 12 Wagner 4 male patients, adjusted for age, BMI, and HbA1c). All three MAGs retained the same direction of effect as in the full cohort: *E. coli* remained significantly enriched in Wagner 4 (coefficient +2.67, *P* = 0.040) and *Collinsella* sp. [S028_bin.32] remained significantly depleted (coefficient −2.84, *P* = 0.048), while *Collinsella* sp905214615 showed the same depleted direction but fell to a non-significant trend (coefficient −3.07, *P* = 0.092) (**Supplementary Table 16**), consistent with reduced statistical power at *n* = 26 rather than a change in direction. Because only one Wagner 4 patient was female, a female-restricted comparison is not statistically meaningful at this sample size. Because age- and sex-dependent gut microbiome patterns have been reported in human adults (25), we cannot fully exclude residual sex-related confounding beyond what covariate adjustment and this sensitivity analysis capture, and adequately powered recruitment across sex strata in advanced DFU remains an important goal for future cohorts.

The cross-sectional design cannot establish whether the *E. coli*–*Collinsella* signal precedes, accompanies or results from advanced tissue injury and systemic inflammation. The positive association with DFU duration (**Supplementary Figure 4**) is compatible with a downstream microbial imprint of chronic or advanced disease, as we favor above, but cross-sectional data cannot exclude a contributory role, and only longitudinal sampling can distinguish between these possibilities. Medication and care-setting effects are additional sources of uncertainty: metformin can produce a strong gut microbiome signature in T2D and may confound disease-associated microbial patterns (20), and hospital-related exposures such as diet, mobility and prior healthcare contact beyond the 4-week pre-enrollment antibiotic-free window were not captured in this dataset. With only 43 patients in total and 13 in the Wagner 4 group, the present cohort was not powered for stratified analysis by medication regimen or care setting; formally stratifying by metformin use and systematically capturing hospital-related exposures will require larger, adequately powered cohorts and is an important direction for future validation studies rather than something the current pilot-scale sample could support.

Future work should validate the *E. coli–Collinsella* signal in larger, multicenter cohorts with longitudinal sampling across infection control, revascularization, debridement, amputation and wound healing. Longitudinal stool metagenomics, with wound metagenomics incorporated where feasible, will help determine whether the gut signal tracks clinical trajectory, treatment response, or recovery, while metatranscriptomic, metabolomic, and culture-based studies can test whether the encoded ARG, VF, and respiratory features are active. Such studies will clarify whether this gut signal can support risk stratification, molecular subtyping or mechanistic investigation of advanced DFU—for example, as a non-invasive companion measure alongside CRP and ESR to help flag patients at higher risk of gangrenous progression, pending prospective validation of its predictive value.

## Materials and methods

### Study population and sample collection

This single-center cross-sectional study was approved by the Ethics Committee of the First Affiliated Hospital of Guangxi Medical University (approval number: 2025-S0001) and was conducted in accordance with the Declaration of Helsinki. All participants provided written informed consent before sample collection.

We prospectively enrolled 43 adult patients with T2DM and active DFU who were admitted to the Department of Endocrinology. Inclusion criteria were: (i) a clinical diagnosis of T2DM according to the 2024 American Diabetes Association criteria; (ii) an active foot ulcer with an assigned Wagner grade (1–4) at admission; and (iii) age ≥ 18 years. Exclusion criteria were: (i) systemic antibiotic therapy within 4 weeks before stool collection; (ii) inflammatory bowel disease, gastrointestinal malignancy, or recent gastrointestinal surgery; (iii) probiotic or prebiotic supplementation within 2 weeks before enrollment; and (iv) pregnancy.

Patients were stratified into Wagner 1–3 (*n* = 30; grades 1, 2, and 3) and Wagner 4 (*n* = 13) according to wound classification recorded by an attending podiatric surgeon at admission. Wagner grade 5 patients were excluded because the short preoperative window in this advanced subgroup limited stool collection. Demographic, anthropometric, biochemical, and inflammatory-marker data (**Table 1**) were extracted from electronic hospital records at the time of stool collection. Laboratory values reported below or above assay detection limits (for example, CRP < 10 mg/L or CRP > 140 mg/L) were imputed at the limit-of-detection midpoint or the upper limit, respectively, before non-parametric comparison. Subject identifiers were replaced with study-specific anonymous IDs (S001–S043) before analysis.

Fresh stool samples were collected on admission into DNA/RNA Shield buffer (Zymo Research) and stored at −80°C within 2 h until DNA extraction.

### DNA extraction, library preparation, and shotgun metagenomic sequencing

Genomic DNA was extracted from approximately 200 mg of homogenized stool using a modified bead-beating-based protocol for fecal microbial community DNA extraction (26). DNA concentration, integrity, and purity were assessed using an Agilent 5400 Fragment Analyzer system (Agilent Technologies, Santa Clara, CA, USA).

Sequencing libraries were prepared from qualified genomic DNA according to the standard BGI Genomics library-construction protocol. Briefly, DNA was randomly fragmented to an approximate 350-bp insert size using a Covaris ultrasonicator (Covaris, Woburn, MA, USA), followed by end repair, A-tailing, ligation of platform-specific adapters, AMPure XP bead purification (Beckman Coulter, USA), and PCR enrichment. Library insert size was verified on an Agilent 2100 Bioanalyzer, and library concentration was quantified by quantitative real-time PCR. Qualified libraries were converted into single-stranded circular DNA, amplified into DNA nanoballs by rolling-circle replication, and loaded onto patterned arrays for combinatorial Probe-Anchor Synthesis sequencing.

Paired-end 150-bp sequencing was performed on the DNBSEQ-T7 platform at BGI Genomics (Shenzhen, China) with a target depth of approximately 10 Gb of raw data per sample. Raw reads were validated against the provider-supplied MD5 checksums prior to downstream analysis.

### Read pre-processing and host decontamination

Read quality before trimming was evaluated with FastQC v0.12.1 and aggregated with MultiQC v1.29. Reads were quality-controlled with KneadData v0.6.1, and host-derived reads were removed by Bowtie2 mapping against the human reference genome GRCh38. After quality control and host decontamination, a median of 76.4 million reads (range 61.5–96.8 million) per sample was retained, totalling approximately 3.3 billion clean reads across the 43 samples for contig assembly (**Supplementary Table 17**).

### Metagenomic assembly, binning, and dereplication of metagenome-assembled genomes

Each sample was independently assembled *de novo* using MEGAHIT v1.2.9 (−−k-max 255 −−k-step 12). Assembly statistics were summarized with QUAST v5.2.0. Within-sample contig binning was performed with MetaBAT2 v2.18 (minimum contig length, 1,500 bp), using contig-depth profiles generated by Bowtie2 followed by jgi_summarize_bam_contig_depths. Across the 43 samples, 2,455 candidate bins were recovered.

Genome completeness and contamination were estimated for all candidate bins using CheckM v1.2.5 with the lineage workflow. Candidate bins were retained for dereplication if they met MIMAG-derived medium-quality completeness/contamination thresholds (completeness ≥ 50% and contamination < 10%) (12). Retained bins were dereplicated with dRep v3.6.2 using primary clustering at MASH ANI ≥ 90% and secondary clustering at ANIm ≥ 95%. Genome quality was subsequently summarized using completeness/contamination thresholds: medium-quality MAGs were defined as completeness ≥ 50% and contamination < 10%, and high-completeness/low-contamination MAGs as completeness ≥ 90% and contamination < 5%. This yielded 440 dereplicated MAGs for the reference catalog. Taxonomic annotation of the 440 MAGs was performed with GTDB-Tk v2.3.2 (release 220); when species-level annotation was unavailable, the lowest-rank classification was retained (for example, Genus sp. [bin_id]).

### MAG-level quantification and taxonomic abundance profiling

Read abundances against the 440 dereplicated MAGs were quantified with CoverM v0.7.0 in genome mode (minimap2-sr mapper, minimum read identity 95%; methods: relative_abundance and count). Output bin IDs were aligned to GTDB-Tk classifications (**Supplementary Table 1**) and reprocessed in Python (pandas) to assign unique MAG-level labels for species represented by multiple bins [for example, *Bacteroides fragilis* (MAG-A) and (MAG-B)]. These labels denote distinct MAGs of the same species and do not imply strain-level genomic differentiation. For analyses at higher taxonomic ranks, MAG-level relative abundance was aggregated to species and genus levels by summing within-rank abundance.

### Functional annotation of MAGs: ARGs, virulence factors, and metabolic themes

Antibiotic resistance genes (ARGs) were identified by running Resistance Gene Identifier (RGI v5.2.0) against CARD v4.0.1 in contig-input mode with BLAST as the alignment tool; only Perfect or Strict hits were retained for downstream analysis. Virulence factors (VFs) were identified with ABRicate v1.2.0 against VFDB (≥ 75% identity and ≥ 60% coverage). Because ABRicate operates on contigs rather than bins, hits were attributed to their parent MAG using a contig-to-bin mapping built from dereplicated bin FASTA files; contigs assigned to more than one bin (*n* = 0 after dereplication) would have been flagged as ambiguous and excluded.

Metabolic functional potential was characterized with DRAM v1.5.0 (annotate followed by distill, default databases). The annotation step used Prodigal-predicted ORFs and returned module-level completeness scores for KEGG-based metabolic themes. DRAM successfully annotated all 440 dereplicated MAGs, producing module-completeness vectors for downstream analyses.

### Microbiome data analysis

Alpha diversity (Inverse Simpson and Shannon indices) was calculated with the vegan package in R, and group differences were evaluated with two-sided Wilcoxon rank-sum tests. Beta-diversity was assessed using Bray-Curtis dissimilarity, visualized by principal coordinate analysis (PCoA) with vegan and ggplot2, and tested by PERMANOVA (adonis2, 999 permutations). Population-level composition shifts among the most abundant MAGs were visualized as Sankey-style plots with ggalluvial.

Differential abundance between Wagner 4 and Wagner 1–3 patients was assessed with MaAsLin2 using TSS normalization and LOG transformation, both with covariate adjustment (main model: age, sex, BMI, and HbA1c) and without covariate adjustment. Concordance in effect direction between the two models was used as a robustness criterion for downstream intersection-based prioritization. As a sensitivity analysis addressing the sex imbalance between groups (**Table 1**), the three-MAG MaAsLin2 model was additionally re-fit in male patients only (14 Wagner 1–3 vs. 12 Wagner 4), with sex dropped from the covariate set and age, BMI, and HbA1c retained.

To identify MAGs that were stably informative under sampling variation, we trained a class-balanced RF classifier (R package randomForest, 500 trees, balanced sampling to the minority class) under a fivefold stability-selection scheme implemented with caret::createFolds. MAGs selected by Boruta-style mean-decrease-Gini ranking in at least three of five folds were retained as RF-selected MAGs. Feature contributions were interpreted with SHAP (SHapley Additive exPlanations) values computed by fastshap. Receiver-operating characteristic (ROC) curves were drawn, and pairwise AUCs were compared by DeLong’s test using the pROC package.

The three-MAG intersection was defined as RF-selected MAGs that also reached MAG-level MaAsLin2 *P* < 0.10 and shared the same effect direction in the unadjusted and covariate-adjusted models. The 24-MAG and three-MAG RF models were evaluated under identical training protocols and standard, non-nested fivefold cross-validation within the discovery cohort.

Functional annotations were summarized per MAG. For visual context, ARG and VF loads were plotted across all other catalog MAGs, RF-selected MAGs outside the three-MAG intersection, and these three MAGs, with Wilcoxon comparisons between the three MAGs and all other catalog MAGs. DRAM theme completeness was compared descriptively between the three MAGs and all other catalog MAGs because formal within-set testing was not meaningful for a three-genome set. These *P*-values were treated as exploratory summaries rather than formal enrichment statistics. Multiple testing across MAG-level MaAsLin2 results was controlled with the Benjamini-Hochberg procedure; both nominal *P*-values, used for the intersection definition threshold above, and BH-adjusted q values are reported (**Supplementary Table 3**).

Associations between the E. coli and Collinsella MAGs and clinical variables were assessed using log10-transformed MAG relative abundance with a cohort-wide pseudocount equal to half the minimum positive abundance. Spearman correlations were calculated in the pooled cohort and within Wagner strata when at least 10 complete observations were available. A composite *E. coli*–*Collinsella* abundance score was defined as z (*E. coli*) minus the mean z-transformed abundance of the two *Collinsella* MAGs. For covariate-adjusted analyses, inverse-normal-transformed clinical variables were regressed on z-scored MAG abundance or the composite score in linear models adjusted for age, sex, BMI, and HbA1c, with an additional model including Wagner group as a sensitivity analysis. The Wagner grade itself was not fitted as an adjusted clinical outcome because it defined the comparison groups.

Continuous baseline characteristics (**Table 1**) were summarized as mean ± s.d. and compared by Welch’s two-sided t-test, or as median [IQR] and compared by two-sided Wilcoxon rank-sum test, depending on the Shapiro-Wilk normality test. Categorical variables were compared by Fisher’s exact test when any expected count was < 5 and by χ*²* tests otherwise. Forest plots, heatmaps, ROC curves, UpSet plots, Venn diagrams and multi-panel figures were generated using ggplot2, pheatmap, UpSetR, ggvenn and patchwork in R, with final layouts assembled in Adobe Illustrator.

## Data Availability

Raw metagenomic sequencing reads have been deposited in the NCBI Sequence Read Archive under accession PRJNA1471479, and analysis scripts are available upon request from the corresponding author.
