# Supplementary material for: Genome-resolved gut metagenomics identifies an Escherichia coli-Collinsella signature associated with Wagner 4 gangrenous diabetic foot ulcers
Source: Front Immunol. 2026 Sep 3;17:1893357. doi: 10.3389/fimmu.2026.1893357 (PMC13581658; doi:10.3389/fimmu.2026.1893357)
Supplement: Supplementary file 1 [file DataSheet1.zip › TableS17.docx]

# Supplementary Table. Software, database versions, source repositories and original references

*All tool versions were verified directly on the analysis server. Source repository links point to the exact tool used; original reference links point to the DOI of the paper describing the method/database.*

| **Software / database** | **Version** | **Source repository** | **reference** |
| --- | --- | --- | --- |
| FastQC | v0.12.1 | [GitHub](https://github.com/s-andrews/FastQC) | Andrews S. FastQC: A Quality Control Tool for High Throughput Sequence Data (software). Babraham Bioinformatics; 2010. [Link](https://www.bioinformatics.babraham.ac.uk/projects/fastqc/) |
| MultiQC | v1.29 | [GitHub](https://github.com/MultiQC/MultiQC) | Ewels P, Magnusson M, Lundin S, Käller M. MultiQC: summarize analysis results for multiple tools and samples in a single report. Bioinformatics. 2016;32(19):3047–3048. <https://doi.org/10.1093/bioinformatics/btw354> |
| KneadData | v0.6.1 | [GitHub](https://github.com/biobakery/kneaddata) | McIver LJ, Abu-Ali G, Franzosa EA, et al. bioBakery: a meta'omic analysis environment. Bioinformatics. 2018;34(7):1235–1237. <https://doi.org/10.1093/bioinformatics/btx754> |
| Bowtie2 | 2.5.4 | [GitHub](https://github.com/BenLangmead/bowtie2) | Langmead B, Salzberg SL. Fast gapped-read alignment with Bowtie 2. Nat Methods. 2012;9(4):357–359. <https://doi.org/10.1038/nmeth.1923> |
| MEGAHIT | v1.2.9 | [GitHub](https://github.com/voutcn/megahit) | Li D, Liu CM, Luo R, Sadakane K, Lam TW. MEGAHIT: an ultra-fast single-node solution for large and complex metagenomics assembly via succinct de Bruijn graph. Bioinformatics. 2015;31(10):1674–1676. <https://doi.org/10.1093/bioinformatics/btv033> |
| QUAST | v5.2.0 | [GitHub](https://github.com/ablab/quast) | Gurevich A, Saveliev V, Vyahhi N, Tesler G. QUAST: quality assessment tool for genome assemblies. Bioinformatics. 2013;29(8):1072–1075. <https://doi.org/10.1093/bioinformatics/btt086> |
| MetaBAT2 | v2.18 | [Bitbucket](https://bitbucket.org/berkeleylab/metabat) | Kang DD, Li F, Kirton E, et al. MetaBAT 2: an adaptive binning algorithm for robust and efficient genome reconstruction from metagenome assemblies. PeerJ. 2019;7:e7359. <https://doi.org/10.7717/peerj.7359> |
| Samtools | 1.23 | [GitHub](https://github.com/samtools/samtools) | Danecek P, Bonfield JK, Liddle J, et al. Twelve years of SAMtools and BCFtools. Gigascience. 2021;10(2):giab008. <https://doi.org/10.1093/gigascience/giab008> |
| CheckM | v1.2.5 | [GitHub](https://github.com/Ecogenomics/CheckM) | Parks DH, Imelfort M, Skennerton CT, Hugenholtz P, Tyson GW. CheckM: assessing the quality of microbial genomes recovered from isolates, single cells, and metagenomes. Genome Res. 2015;25(7):1043–1055. <https://doi.org/10.1101/gr.186072.114> |
| dRep | v3.6.2 | [GitHub](https://github.com/MrOlm/drep) | Olm MR, Brown CT, Brooks B, Banfield JF. dRep: a tool for fast and accurate genomic comparisons that enables improved genome recovery from metagenomes through de-replication. ISME J. 2017;11(12):2864–2868. <https://doi.org/10.1038/ismej.2017.126> |
| GTDB-Tk | v2.3.2 (R220) | [GitHub](https://github.com/Ecogenomics/GTDBTk) | Chaumeil PA, Mussig AJ, Hugenholtz P, Parks DH. GTDB-Tk v2: memory friendly classification with the Genome Taxonomy Database. Bioinformatics. 2022;38(23):5315–5316. <https://doi.org/10.1093/bioinformatics/btac672> |
| CoverM | v0.7.0 | [GitHub](https://github.com/wwood/CoverM) | Woodcroft BJ, et al. CoverM (software). No peer-reviewed publication; cite the software repository directly. [Link](https://github.com/wwood/CoverM) |
| RGI (Resistance Gene Identifier) | v5.2.0 | [GitHub](https://github.com/arpcard/rgi) | Alcock BP, Huynh W, Chalil R, et al. CARD 2023: expanded curation, support for machine learning, and resistome prediction at the Comprehensive Antibiotic Resistance Database. Nucleic Acids Res. 2023;51(D1):D690–D699. <https://doi.org/10.1093/nar/gkac920> |
| CARD | v4.0.1 | [Database homepage](https://card.mcmaster.ca/) | Alcock BP, Huynh W, Chalil R, et al. CARD 2023: expanded curation, support for machine learning, and resistome prediction at the Comprehensive Antibiotic Resistance Database. Nucleic Acids Res. 2023;51(D1):D690–D699. <https://doi.org/10.1093/nar/gkac920> |
| ABRicate | v1.2.0 | [GitHub](https://github.com/tseemann/abricate) | Seemann T. ABRicate (software). No peer-reviewed publication; cite the software repository directly. [Link](https://github.com/tseemann/abricate) |
| VFDB | as distributed with ABRicate v1.2.0* | [Database homepage](http://www.mgc.ac.cn/VFs/) | Liu B, Zheng D, Zhou S, Chen L, Yang J. VFDB 2022: a general classification scheme for bacterial virulence factors. Nucleic Acids Res. 2022;50(D1):D912–D917. <https://doi.org/10.1093/nar/gkab1107> |
| DRAM | v1.5.0 | [GitHub](https://github.com/WrightonLabCSU/DRAM) | Shaffer M, Borton MA, McGivern BB, et al. DRAM for distilling microbial metabolism to automate the curation of microbiome function. Nucleic Acids Res. 2020;48(16):8883–8900. <https://doi.org/10.1093/nar/gkaa621> |

**VFDB does not carry an independent release version the way CARD does; it is whatever build ships with the installed ABRicate version.*

*CoverM and ABRicate have no peer-reviewed methods paper as of this writing; per standard practice for such tools, we cite the software repository itself rather than fabricate a journal reference.*
