# Supplementary material for: Genome-resolved gut metagenomics identifies an Escherichia coli-Collinsella signature associated with Wagner 4 gangrenous diabetic foot ulcers
Source: Front Immunol. 2026 Sep 3;17:1893357. doi: 10.3389/fimmu.2026.1893357 (PMC13581658; doi:10.3389/fimmu.2026.1893357)
Supplement: Supplementary file 1 [file DataSheet1.zip › Table1.docx]

## 1.1. Table 1. Baseline characteristics of the study cohort by Wagner severity group.

| **Characteristic** | **Wagner 1–3 (n = 30)** | **Wagner 4 (n = 13)** | **P value** | **Test** |
| --- | --- | --- | --- | --- |
| **── Demographics ──** |  |  |  |  |
| Age, years | 62.0 [58.0, 67.0] | 62.0 [61.0, 70.0] | 0.496 | Wilcoxon |
| Sex, n (%) |  |  | 0.013 | chi-square |
| Male | 14 (46.7) | 12 (92.3) |  |  |
| Female | 16 (53.3) | 1 (7.7) |  |  |
| Ethnicity, n (%) |  |  | 0.460 | Fisher |
| Han | 23 (76.7) | 8 (61.5) |  |  |
| Minority | 7 (23.3) | 5 (38.5) |  |  |
| **── Anthropometric ──** |  |  |  |  |
| Height, cm | 161.6 ± 7.9 | 164.9 ± 4.8 | 0.096 | t-test |
| Weight, kg | 57.5 [52.3, 69.6] | 64.0 [59.4, 73.0] | 0.109 | Wilcoxon |
| BMI, kg/m² | 22.4 [21.0, 25.6] | 23.0 [21.7, 25.7] | 0.513 | Wilcoxon |
| **── Diabetes / DFU history ──** |  |  |  |  |
| DM duration, years | 9.0 [3.0, 14.5] | 9.0 [5.0, 20.0] | 0.427 | Wilcoxon |
| DFU duration, days | 30.0 [8.5, 52.5] | 60.0 [15.0, 60.0] | 0.161 | Wilcoxon |
| Prior DFU history, n (%) |  |  | 1.000 | Fisher |
| No | 23 (76.7) | 10 (76.9) |  |  |
| Yes | 7 (23.3) | 3 (23.1) |  |  |
| HbA1c, % | 8.5 [6.9, 9.8] | 8.7 [6.4, 10.4] | 1.000 | Wilcoxon |
| **── Inflammatory markers ──** |  |  |  |  |
| Peak temperature, °C | 36.8 [36.6, 37.0] | 36.8 [36.7, 36.9] | 0.800 | Wilcoxon |
| WBC, ×10⁹/L | 8.6 [6.6, 10.2] | 10.6 [7.7, 12.5] | 0.135 | Wilcoxon |
| Neutrophil, % | 0.7 ± 0.1 | 0.7 ± 0.1 | 0.110 | t-test |
| CRP, mg/L | 5.0 [5.0, 29.5] | 44.4 [25.2, 100.0] | 0.005 | Wilcoxon |
| PCT, ng/mL | 0.06 [0.03, 0.55] | 0.16 [0.06, 0.47] | 0.126 | Wilcoxon |
| ESR, mm/h | 58.0 [33.0, 80.0] | 92.0 [68.0, 105.0] | 0.003 | Wilcoxon |
| **── Biochemistry ──** |  |  |  |  |
| ALT, U/L | 15.0 [11.0, 22.0] | 17.0 [15.0, 28.0] | 0.340 | Wilcoxon |
| AST, U/L | 17.0 [13.0, 22.0] | 19.0 [16.0, 32.0] | 0.470 | Wilcoxon |
| Albumin, g/L | 35.9 ± 8.3 | 31.9 ± 6.1 | 0.087 | t-test |
| Urea, mmol/L | 6.1 [5.0, 8.7] | 7.4 [5.1, 9.8] | 0.341 | Wilcoxon |
| Creatinine, µmol/L | 83.5 [68.2, 109.2] | 108.0 [81.0, 132.0] | 0.116 | Wilcoxon |
| Hemoglobin, g/L | 116.5 [95.1, 125.0] | 86.0 [82.0, 100.9] | 0.022 | Wilcoxon |
| Platelets, ×10⁹/L | 320.7 ± 128.3 | 383.6 ± 179.6 | 0.268 | t-test |
| Total cholesterol, mmol/L | 4.12 [3.39, 5.17] | 3.31 [2.79, 3.41] | 0.111 | Wilcoxon |
| Triglycerides, mmol/L | 1.15 [0.86, 1.78] | 1.14 [0.88, 1.36] | 0.817 | Wilcoxon |
| HDL-C, mmol/L | 0.95 ± 0.30 | 0.90 ± 0.29 | 0.589 | t-test |
| LDL-C, mmol/L | 2.41 ± 0.84 | 1.82 ± 0.98 | 0.076 | t-test |
| Continuous variables: mean ± SD (Welch's t-test) if normally distributed (Shapiro-Wilk p ≥ 0.05 in both groups), otherwise median [Q1, Q3] (Wilcoxon rank-sum test). | | | | |
| Categorical variables: n (%); Fisher exact test (any expected cell < 5) or chi-square test. | | | | |
| CRP values reported as <10 mg/L by the laboratory were imputed at 5 mg/L (limit-of-detection midpoint); one value reported as >140 mg/L was set to 140 mg/L. | | | | |
| DM, diabetes mellitus; DFU, diabetic foot ulcer; HbA1c, glycated hemoglobin; WBC, white blood cell count; CRP, C-reactive protein; PCT, procalcitonin; ESR, erythrocyte sedimentation rate. | | | | |
